# Antioxidant Effects of Grape Vine Cane Extracts from Different Chinese Grape Varieties on Edible Oils

**DOI:** 10.3390/molecules190915213

**Published:** 2014-09-23

**Authors:** Zhuo Min, Zemei Guo, Kai Wang, Ang Zhang, Hua Li, Yulin Fang

**Affiliations:** 1 College of Enology, Northwest A&F University, Yangling 712100, Shaanxi, China; 2 Shaanxi Engineering Research Center for Viti-Viniculture, Yangling 712100, Shaanxi, China

**Keywords:** grape vine cane, extracts, edible oils, antioxidant

## Abstract

This study involved the determination of the peroxide value (POV) as a measure of the resistance of the oxidation of edible oil with grape vine cane additives to assess their antioxidation potential. The study demonstrated that grape extracts of canes could effectively inhibit the lipid oxidation of edible oils and that this ability varied significantly due to the different extraction solvents employed, as well as to the different varieties of canes used. Lipid oxidation of edible oils was significantly reduced under an accelerated storage condition of 70 ± 1 °C in the presence of Vitamin C (VC), which was chosen as a synergist of grape vine cane extract. A 4:1 ratio of Victoria Blanc-ethyl acetate fraction (EAF) and VC led to a significant lowering of the peroxide value and indicated a better antioxidant effect. Thus, these results indicated that some varieties of grape vine cane extracts could be applied as natural antioxidants for elevation of the quality of edible oils in the food industry.

## 1. Introduction

Edible oils are known to supply energy and are a source of some essential fatty acids, which act as carriers of fat-soluble vitamins VA, VD, VE, and VK along with other fat-soluble nutrients, making them highly significant for human health. Generally, edible oils containing a high measure of unsaturated fatty acids have been found to undergo oxygenation easily upon exposure to light, heat, oxygen, and metal ions, or a combination of these conditions [1]. Thus, because of oxygenation, their nutritive value reduces, and they produce offensive smells and sometimes even generate harmful noxious substances. However, lipid peroxidation may be retarded or restrained effectively by adding synthetic [2] or natural antioxidants to the edible oils [3,4,5]. 

At present, the commonly used synthetic antioxidants include butylated hydroxytoluene (BHT), butylated hydroxyanisole (BHA), tert-butyl hydroquinone (TBHQ), and propyl gallate (PG). Many countries have limited or prohibited the use of these synthetic antioxidants owing to some toxicity, which has been implicated as a trigger for cancer, thus paving the way for natural antioxidants, which contain phenolic compounds or antioxidant activities attributed to specific peptides, phytic acid, pigments like anthocyanins, and many polysaccharides. Natural antioxidants, which are currently in use, mainly include vitamin E, tea polyphenol (TP), nordihydroguaiaretic acid (NDGA), and rosemary. Furthermore, some fruits, vegetables, spices, Chinese herbal medicines, and many other plants contain natural antioxidants, [6] of which many have demonstrated inhibitory action towards lipid peroxidation. Some displayed abilities similar to TBHQ [7], some to BHT [8], and others to BHA–BHT [9], and were considered to be safe, healthy, and harmless [10]. 

Grape polyphenols, which are extracted from grapes and their branches, is a generic term involving phenolic compounds from grapes, which possess multiple hydroxyls. They comprise important secondary metabolites of grapes and their antioxidant ability is well understood [11]. Amongst them, particularly, proanthocyanidins are known to be 20 times more potent than vitamin E and 50 times more potent than vitamin C [12]. Grape polyphenols are known to protect the liver, act as detoxifiers, and they show bacteriostatic, antitumor [13,14], and anti-allergic properties. Moreover, they have been used for skin protection and for preventing angiocardiopathy [15]. For instance, resveratrol, an ingredient of red wine, has been shown to resist oxidation and scavenge generated radicals well, and consequently, it prevents damage to the stomach caused by oxidation in the enterocoelia [16]. Moreover, it has also been confirmed that grape seed extract (GSE) is a very effective inhibitor of lipid oxidation and can be recommended as a potential natural antioxidant for the vegetal oil industry [17], and that the polyphenolic fractions from murta leaves can improve the oxidative stability of micro-encapsulated linseed oil [18]. Therefore, grape polyphenols have found applications in the fields of health care, food, chemical, and environmental protection. 

In this study, polyphenols were extracted with different organic solvents from grape canes of various varieties, and their antioxidation in edible oils was analyzed, for the purpose of exploring an alternative natural antioxidant, which may be preferred for use to elevate the quality of edible oils in the food industries, as well as for determining the optimal ratio of grape vine cane extracts with Vc, a well-known synergist.

## 2. Results and Discussion

### 2.1. Antioxidant Activity Studies of Grape Vine Cane Extracted with Different Solvents

The antioxidant potentials reflected from the peroxide value (POV) are displayed in Table 1, Table 2, Table 3 and Table 4. The values increased by different degrees on increasing the storage period, indicating that all the extracts had antioxidant effects on lard oil. Among the samples treated with grape vine cane extracts in ME, “Shuangyou” exhibited minimum POVs at 4, 6 and 8 days. Remarkably, the blank showed a higher (*p* *<* 0.05) POV on increasing the storage period from the fourth to the sixth day as compared to the treated samples. TBHQ treated samples showed significantly (*p <* 0.05) lower POVs than others on increasing the storage period from the fourth to the sixth day (Table 1). 

**Table 1:** Peroxide value (POV) (meqO_2_/kg sample) of edible oil samples treated with tert-butyl hydroquinone (TBHQ) and grape vine cane extracts by the methanol antioxidation test at 70 ± 1 °C, kept in the dark.

Treatment	Storage Period (Days)				
	0	2	4	6	8
*Vitis heyneana*	1.30 ± 0.05	3.22 ^b^ ± 0.26	6.33 ^e^ ± 0.81	26.67 ^c^ ± 2.14	54.70 ^b,c^ ± 4.11
Baiyu	1.30 ± 0.05	3.21 ^b^ ± 0.24	7.29 ^c^ ± 0.59	23.56 ^g^ ± 2.03	46.58 ^e^ ± 4.48
Tchervine muscat	1.30 ± 0.05	2.60 ^d^ ± 0.21	5.23 ^f^ ± 0.52	20.51 ^i^ ± 2.09	53.44 ^c,d^ ± 3.47
Bei binghong	1.30 ± 0.05	1.84 ^g^ ± 0.17	4.82 ^g^ ± 0.48	17.32 ^k^ ± 1.22	44.60 ^e,f^ ± 4.44
FOX	1.30 ± 0.05	2.54 ^d^ ± 0.12	5.04 ^f,g^ ± 0.54	18.82 ^j^ ± 2.07	52.20 ^d^ ± 3.23
Junzi	1.30 ± 0.05	3.55 ^a^ ± 0.26	7.28 ^c^ ± 0.82	25.30 ^d,e^ ±1.85	50.36 ^d^ ± 4.02
Granoir	1.30 ± 0.05	2.30 ^e^ ± 0.15	6.24 ^e^ ± 0.77	21.65 ^h^ ± 1.98	43.04 ^f^ ± 4.07
Conquister	1.30 ± 0.05	3.35 ^b^ ± 0.32	10.23 ^b^ ± 1.05	28.23 ^b^ ± 2.22	55.50 ^b^ ± 5.02
Shuangyou	1.30 ± 0.05	2.05 ^f^ ± 0.27	4.30 ^h^ ± 0.62	14.35 ^l^ ± 1.26	30.85 ^h^ ± 2.18
Beta	1.30 ± 0.05	2.43 ^d,e^ ± 0.22	4.73 ^g^ ± 0.54	16.28 ^k^ ± 1.71	38.56 ^g^ ± 2.83
Vidal	1.30 ± 0.05	2.80 ^c^ ± 0.11	6.51 ^d,e^ ± 0.66	23.87 ^f,g^ ±1.82	45.30 ^e^ ± 2.39
Cabernet sauvignon	1.30 ± 0.05	2.78 ^c^ ± 0.03	6.68 ^d^ ± 0.53	26.45 ^c,d^ ±2.81	49.62 ^d^ ± 3.24
Shuanghong	1.30 ± 0.05	2.80 ^c^ ± 0.10	4.70 ^g^ ± 0.46	24.71 ^e,f^ ±2.63	55.54 ^b^ ± 5.42
TBHQ	1.30 ± 0.05	1.88 ^f,g^ ± 0.09	2.08 ^i^ ± 0.31	2.16 ^m^ ± 0.13	3.82 ^i^ ± 0.18
Blank test	1.30 ± 0.05	3.67 ^a^ ± 0.53	14.32 ^a^ ± 1.12	45.66 ^a^ ± 5.84	100.46 ^a^ ± 6.76

Mean of three values of replicates have been presented with their standard deviation. All the mean values, denoted with different superscript letters within a column (a–m), are significantly different at *p <* 0.05.

**Table 2:** POV (meqO_2_/kg sample) of edible oils samples treated with TBHQ and grape polyphenol extracts from canes with petroleum ether antioxidation test at 70 ± 1 °C, kept in the dark.

Treatment	Storage Period (Days)					
	0	3	5	7	9	11
FOX	1.25 ± 0.03	3.80 ^c^ ± 0.23	7.31 ^f^ ± 0.72	23.29 ^f^ ± 2.23	66.41 ^d^ ± 5.51	84.68 ^e^ ± 6.03
Cabernet sauvignon	1.25 ± 0.03	3.32 ^e^ ± 0.13	8.28 ^e^ ± 0.52	25.98 ^e^ ± 4.02	61.94 ^f^ ± 5.61	94.37 ^d^ ± 11.69
Vidal	1.25 ± 0.03	3.50 ^d^ ± 0.32	9.28 ^c^ ± 0.15	34.06 ^d^ ± 3.24	72.55 ^c^ ± 8.42	98.87 ^c^ ± 7.02
Shuanghong	1.25 ± 0.03	3.82 ^c^ ± 0.41	8.76 ^d^ ± 0.13	36.36 ^c^ ± 2.63	63.11 ^e^ ± 6.13	107.20 ^b^ ± 8.10
Beta	1.25 ± 0.03	3.74 ^c^ ± 0.54	6.95 ^g^ ± 0.12	21.64 ^g^ ± 3.01	59.64 ^g^ ± 5.13	83.31 ^e^ ± 5.04
Junzi	1.25 ± 0.03	4.27 ^b^ ± 0.53	10.24 ^b^ ± 1.61	38.38 ^b^ ± 4.02	73.53 ^b^ ± 7.22	93.58 ^d^ ± 7.62
Victoria Blanc	1.25 ± 0.03	1.36 ^g^ ± 0.13	3.28 ^i^ ± 0.34	7.68 ^i^ ± 1.04	20.28 ^i^ ± 2.80	39.89 ^g^ ± 4.22
*Vitis heyneana*	1.25 ± 0.03	2.29 ^f^ ± 0.23	4.92 ^h^ ± 0.44	10.42 ^h^ ± 1.01	42.70 ^h^ ± 4.23	79.90 ^f^ ± 5.71
Blank test	1.25 ± 0.03	6.07 ^a^ ± 0.50	15.09 ^a^ ± 1.76	47.20 ^a^ ± 5.31	104.08 ^a^ ± 12.4	150.00 ^a^ ± 12.5
TBHQ	1.25 ± 0.03	1.29 ^g^ ± 0.04	2.09 ^j^ ± 0.22	2.46 ^j^ ± 0.42	3.14 ^j^ ± 0.32	3.90 ^h^ ± 0.43

Mean of three values of replicates presented with their standard deviation. Mean values that have been denoted with different superscript letters within a column (a–j) are significantly different at *p* < 0.05.

**Table 3:** POV (meqO_2_/kg sample) of edible oils samples treated with TBHQ and grape polyphenol extracts from canes with chloroform antioxidation test at 70 ± 1 °C, kept in the dark.

Treatment	Storage Period (Days)					
	0	3	5	7	9	11
*Vitis heyneana*	1.25 ± 0.07	1.88 ^c^ ± 0.12	4.36 ^c^ ± 0.09	10.40 ^c^ ± 1.49	43.90 ^d^ ± 3.33	73.20 ^d^ ± 8.21
Baiyu	1.25 ± 0.07	1.69 ^d^ ± 0.08	3.42 ^d^ ± 0.13	8.82 ^e^ ± 0.92	41.33 ^e^ ± 4.19	79.65 ^b^ ± 6.47
Cabernet sauvignon	1.25 ± 0.07	1.78c ^d^ ± 0.11	4.31 ^c^ ± 0.16	9.84 ^d^ ± 0.95	46.62 ^c^ ± 3.76	80.67 ^b^ ± 8.09
FOX	1.25 ± 0.07	1.58 ^d^ ± 0.21	3.56 ^d^ ± 0.22	7.16 ^f^ ± 1.36	31.04 ^g^ ± 2.98	57.82 ^e^ ± 10.0
Beta	1.25 ± 0.07	1.65 ^d^ ± 0.07	3.18 ^d^ ± 0.32	8.92 ^d,e^ ± 0.63	34.64 ^f^ ± 6.0	70.67 ^d^ ± 3.33
Junzi	1.25 ± 0.07	2.39 ^b^ ± 0.22	6.06 ^b^ ± 1.10	21.29 ^b^ ± 2.47	59.82 ^b^ ± 3.19	76.07 ^c^ ± 4.62
Blank test	1.25 ± 0.07	6.07 ^a^ ± 0.84	15.09 ^a^ ± 2.02	47.20 ^a^ ± 5.12	104.08 ^a^ ± 8.89	150.00 ^a^ ±11.8
TBHQ	1.25 ± 0.07	1.29 ^e^ ± 0.14	2.09 ^e^ ± 0.12	2.46 ^g^ ± 0.22	3.14 ^h^ ± 0.12	3.90 ^f^ ± 0.43

Mean of three values of replicates presented along with their standard deviation. Mean values that have been denoted with different superscript letters within a column (a–h) are significantly different at *p* < 0.05.

**Table 4:** POV (meqO_2_/kg sample) of edible oils samples treated with TBHQ and grape polyphenol extracts from canes with ethyl acetate antioxidation test at 70 ± 1 °C, kept in the dark.

Treatment	Storage Period (Days)				
	0	2	4	6	8
Cabernet sauvignon	2.20 ± 0.16	2.80 ^d,e^ ± 0.22	5.93 ^g^ ± 0.62	12.48 ^h^ ± 1.42	31.10 ^g^ ± 3.12
Junzi	2.20 ± 0.16	3.04 ^b^ ± 0.09	7.80 ^d^ ± 0.67	21.64 ^c^ ± 2.03	41.24 ^d^ ± 1.93
FOX	2.20 ± 0.16	2.76 ^d,e^ ± 0.08	8.28 ^c^ ± 0.84	24.45 ^b^ ± 2.33	49.82 ^b^ ± 3.42
Bei binghong	2.20 ± 0.16	2.70 ^e^ ± 0.32	4.62 ^h^ ± 0.63	18.08 ^e^ ± 1.52	44.20 ^c,d^ ± 3.78
Vidal	2.20 ± 0.16	2.74 ^d,e^ ± 0.31	5.08 ^h^ ± 0.75	12.14 ^h^ ± 1.61	35.00 ^f^ ± 1.87
Beta	2.20 ± 0.16	2.72 ^d,e^ ± 0.09	6.38 ^f^ ± 0.23	16.48 ^f^ ± 2.02	38.76 ^e^ ± 2.47
*Vitis heyneana*	2.20 ± 0.16	2.83 ^c,d^ ± 0.22	9.52 ^b^ ± 1.22	20.86 ^d^ ± 1.67	39.70 ^e^ ± 2.75
Baiyu	2.20 ± 0.16	2.93 ^c^ ± 0.25	4.70 ^h^ ± 0.44	7.28 ^j^ ± 0.63	24.92 ^h^ ± 1.92
Shuangyou	2.20 ± 0.16	2.81 ^d,e^ ± 0.12	7.69 ^d^ ± 0.62	20.20 ^d^ ± 2.72	46.05 ^c^ ± 4.33
Shuanghong	2.20 ± 0.16	3.00 ^b^ ± 0.34	6.72 ^e^ ± 0.73	14.71 ^g^ ± 1.92	35.59 ^f^ ± 4.15
Conquister	2.20 ± 0.16	2.42 ^f^ ± 0.07	5.02 ^h^ ± 0.21	10.74 ^i^ ± 0.55	30.62 ^g^ ± 4.45
Victoria Blanc	2.20 ± 0.16	2.28 ^f^ ± 0.06	3.82 ^i^ ± 0.22	7.13^j^ ± 0.66	18.54 ^i^ ± 1.09
Tchervine muscat	2.20 ± 0.16	2.98 ^b^ ± 0.24	3.94 ^i^ ± 0.19	10.50 ^i^ ± 0.35	26.44 ^h^ ± 2.18
Blank test	2.20 ± 0.16	3.38 ^a^ ± 0.13	14.46 ^a^ ± 1.27	43.66 ^a^ ± 6.03	72.63 ^a^ ± 3.58
TBHQ	1.25 ± 0.07	1.29 ^e^ ± 0.14	2.09 ^e^ ± 0.12	2.46 ^g^ ± 0.22	3.14 ^h^ ± 0.12

Mean of three values of replicates presented along with their standard deviation. Mean values that have been denoted with different superscript letters within a column (a–j) are significantly different at *p* < 0.05.

The petroleum ether fraction (PEF) blank was notably (*p <* 0.05) different from other treatments at 3, 5, 7, 9 and 11 days. The Victoria Blanc (VB) also displayed (*p <* 0.05) differences from the control in comparison to values obtained from other natural antioxidants at 5, 7, 9 and 11 days. TBHQ showed significantly (*p <* 0.05) lower POVs than others from the fifth to the eleventh day (Table 2). 

In general, the POV of all edible oil samples did not exhibit a considerable increase (*p* > 0.05) with increasing storage time. Values for the blank and TBHQ showed substantially (*p <* 0.05) higher and lower POVs than the others on increasing the storage period from 3–11 days, and TBHQ (Table 3) and FOX displayed an excellent performance at 5 days. 

The corresponding values for the ethyl acetate fraction (EAF) and the blank were different (*p <* 0.05) from treatments at 2, 4, 6, and 8 days. Victoria Blanc (VB) showed a significantly (*p <* 0.05) lower POV on increasing the storage period to the 6th day as compared to other treated samples (Table 4).

On comprehensive evaluation, the antioxidant effect on lard oil of VB-PEF and VB-EAF was found to be better than that of others. As described by Pazos, the antioxidant effect of fish oil and fish oil-water emulsion was different because of the differing nature of polyphenols. In fish oil, the antioxidation depended on the physical and chemical properties more than on the redox potential of the antioxidant itself [19]. Previously, a similar conclusion was drawn by research on green tea polyphenols [20]. Moreover, the antioxidant effect could be dissimilar due to different grape varieties, for example, the seedless varieties displayed more potency towards restraining lipid peroxidation [21]. Similarly, Victoria Blanc (VB) polyphenols showed a better antioxidant effect in lard oil. However, the POVs for all extracts were higher than TBHQ, a synthetic antioxidant, indicating that the antioxidant effect of TBHQ was the best in the sample group although the tolerance to different materials was different [22]. Thus, at different temperatures, the antioxidant effect of TBHQ, BHT and a new natural antioxidant were determined, and the results showed that the heat stability of TBHQ was the highest [23], consequently implying that this could be the reason for the higher oxidation resistance of TBHQ at the high temperature of 70 ± 1 °C.

### 2.2. Synergistic Effect of Grape Polyphenols with Synergist

**Figure 1:**
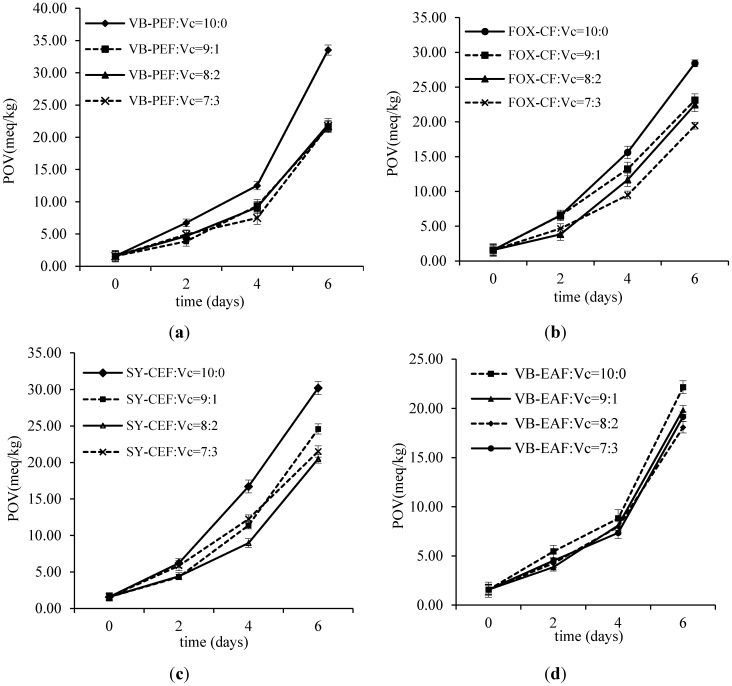
Peroxide value (POV) of edible oils at the ratios of 10:0, 9:1, 4:1, 7:3 of VB-PEF (**a**), FOX-CF (**b**), VB-EAF (**c**), SY-ME (**d**), respectively, with vitamin C, during storage at 70 ± 1 °C for 6 days.

**Figure 2:**
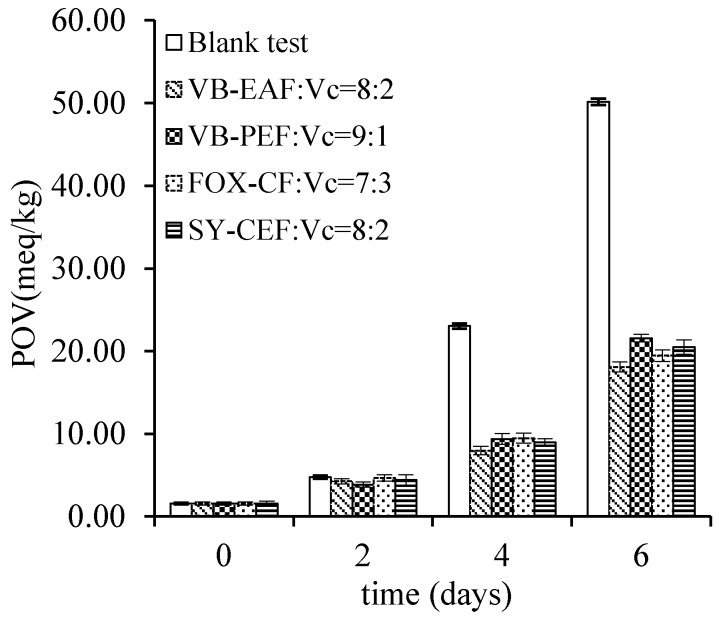
The best synergy of ratios among VB-PEF, FOX-CF, VB-EAF, SY-ME, respectively, with Vc were selected and compared with each other. Storage condition: temperature at 70 ± 1 °C, kept in the dark for 6 days.

In this study, vitamin C was chosen as a synergist for VB-PEF, VB-EAF, FOX-CF, and SY-ME (see Section 3.2 for abbreviations). The ratio of polyphenols and vitamin C was varied as 10:0, 9:1, 4:1, and 7:3 respectively. Vitamin C and polyphenols showed a certain synergistic effect on rap oil. The time of oxidation was extended because of the addition of the synergist and it was compared with that without the synergist; the result showing that the antioxidant effect of VB-EAF was better than that of the others. A comparison of VB-EAF and Vc at a ratio of 4:1 showed a remarkable antioxidant effect (Figure 1 and Figure 2). Vc was found to be related to the antioxidant activity [24], and studies confirmed that it showed good oxidation resistance itself [25]. The total polyphenols of actinidia and Vc showed a high synergistic correlation to oxidation resistance [26]. Vc and VE also had a synergistic effect as an antioxidant [22]; however, only a certain dose of Vc had a synergistic effect with resveratrol [27]. The same applied to grape polyphenols where the synergy was a maximum only at an appropriate ratio with Vc.

## 3. Experimental Section

### 3.1. Materials

Fourteen grape cane samples were employed in this study, namely, five wine grapes “Shuangyou”, “Shuanghong”, and “Beibinghong” of *Vitis amurensis* from Tonghua, Jilin Province, “Junzi” and “Baiyu” of *Vitis davidii* from Chongyi, Jiangxi Province; four cultivars “*Vitis heyneana*” of *Vitis pentagona* from Lantian, Shaanxi Province, “Cabernet Sauvignon”, “Tchervine muscat”, and “Victoria Blanc Blanc” of *Vitis vinifera* from the experimental vineyards of the College of Enology, Northwest A&F University at Yangling, Shaanxi Province. One-year-old canes were collected during the pruning period in 2008. All the chopped cane samples were immediately frozen in liquid nitrogen, freeze-dried and were ground through a 0.5-mm sieve using a domestic electrical grinder (final particle size <0.5 mm). The ground particles were vacuum packed in plastic bags and were stored at −20 °C until extraction.

Fresh pig suet boiled into lard oil by a wet process was obtained from the franchisee of YuRun (Yang Ling, China), and fresh rap oil was procured from an oil mill before the addition of additives. 

All the chemicals and reagents used were of analytical grade. Glacial acetic acid, chloroform, sodium thiosulfate, soluble starch, ascorbic acid, and potassium iodide were procured from BODI Chemical Co., LTD, Shanghai, China. TBHQ was purchased from Sigma Chemical Co., St. Louis, MO, USA.

### 3.2. Solvent Extraction

All the experiments were performed in triplicate. The pulverized grape canes (100 g, dry weight) were exhaustively extracted three times with 1000 mL of acidified methanol solution (1 N HCl/methanol/water, 1/80/19, v/v/v) in a shaking incubator for 24 h at 20 °C. The solvent was separated by vacuum filtration, and the combined supernatants were evaporated in an evaporator at 35 °C, followed by removal of the remaining water by lyophilization to obtain a crude methanolic extract (ME). A known amount of ME was re-suspended in distilled water having a solid-to-liquid ratio of 1:10 (w/v) and partitioned with an equal volume of petroleum ether, chloroform, and ethyl acetate (3 × 1:1, v/v), affording the petroleum ether fraction (PEF), chloroform fraction (CF), and an ethyl acetate fraction (EAF), respectively. The varied cane extracts have been listed according to the extraction solvent used. All the extracts were stored at −20 °C for further analysis.

ME: *Vitis heyneana*, Baiyu, Tchervine muscat, Bei binghong, FOX, Junzi, Granoir, Conquister, Shuangyou (SY), Beta, Vidal, Cabernet Sauvignon, Shuanghong; PEF: FOX, Cabernet Sauvignon, Vidal, Shuanghong, Beta, Junzi, Victoria Blanc (VB), *Vitis heyneana*; Chloroform phase: *Vitis heyneana*, Baiyu, Cabernet Sauvignon, FOX, Beta, Junzi; EAF: Cabernet Sauvignon, Junzi, FOX, Bei binghong, Vidal, Beta, *Vitis heyneana*, Baiyu, Shuangyou (SY), Shuanghong, Conquister, Victoria Blanc (VB), Tchervine Muscat.

### 3.3. Preparation of Reagents

Chloroform-glacial acetic acid solution contained 40 mL of chloroform and 60 mL of glacial acetic acid obtained by shaking the flask until the formation of a homogenous and clear solution. Saturated solutions were prepared in 20% aqueous potassium iodide (10 g of potassium iodide + 5 mL of water) and stored in a brown bottle. Exactly 10 mL of 100 mM stock-form sodium thiosulfate (accurately measured to 0.1 mL) was introduced into a 100-mL volumetric flask and diluted with water to 10 mM working standard of sodium thiosulfate. One mL water was added into 0.5 g of soluble starch to form a paste, which was then poured into 100 mL of boiling water to give a homogeneous solution. A starch indicator having a mass fraction of 0.5% needed to be freshly prepared.

### 3.4. Analytical Methods

The antioxidant activity was expressed as peroxide value (POV). 

The peroxide value (POV) was determined by titration. Dark bottles containing 2–3 g of blended and filtered samples were shaken vigorously after adding 30 mL of chloroform-glacial acetic acid solution and 1 mL of saturated potassium iodide solution. The bottle was placed in the dark for 3 min, after which 50 mL of water was added, vigorously shaken, and immediately titrated with a 10 mM sodium thiosulfate until the colorless solution appeared light yellow. Titration was continued after adding 1 mL of starch indicator until the blue color disappeared. The blank reading was also determined. The titration was carried out manually, in accordance with GB/T5009.37-2003, and the POV was calculated using the following equation:

POV = (V_1_ − V_2_) × C × 0.1269 × 78.8 × 100/m (1)

The volume of the samples titrated with a standard solution of sodium thiosulfate was expressed as V_1_, while V_2_ corresponded to the volume of the sample blank in milliliter (mL). C was the concentration of sodium thiosulfate, and m represented the quality of the samples. The number 0.1269 indicated the quality of iodine, equal to 1.00 mL of standard solution of sodium thiosulfate (Na_2_S_2_O_3_ = 1.00 mol/L); The conversion factor was 78.8.

The POV was expressed as milliequivalents (mequiv) of active oxygen per kilogram of oil.

### 3.5. Sample Preparation for Antioxidant Test in the Dryer under Accelerated Storage Condition

Grape vine cane extracts were added into the edible oils at a proportion of 0.04%. A blank test was also run at the same time for comparison. After blending, edible oils were placed in a drying oven, set at 70 ± 1 °C. Samples were stirred once every 12 h and POVs were determined periodically. The determinations were carried out in triplicate until the critical point of oxidation. According to GB10146-88 standard of hygiene, lard oil’s POV ≤ 16 meg/kg and rap oil’s POV ≤ 12 meg/kg can measure up to the standard of edible oil [28]. 

### 3.6. Synergistic Effect of Grape Vine Cane Extracts with Synergist

Vitamin C was chosen as a synergist, and was added into the edible oils. POVs of edible oils at the ratios of 10:0, 9:1, 4:1, 7:3 of VB-PEF, FOX-CF, VB-EAF, SY-ME, respectively, with vitamin C, during storage at 70 ± 1 °C for 6 days, were determined.

## 4. Conclusions

From the above study, it can be concluded that grape vine cane extracts have an antioxidant effect on edible oils; however, oxidation resistance can be affected by the varieties and the extraction solvent. VB-EAF and VB-PEF were found to be the most promising but their performance could not surpass that of the synthetic antioxidant TBHQ. Despite this, natural antioxidants may be preferred on grounds of safety and economy, and need to be further researched and developed. Grape vine canes showed a synergistic effect with Vc and can enhance oxidation resistance to the oils only if the ratio of grape vine cane extracts and Vc is appropriate.

## Abbreviations

POV: Peroxide value
EAF: Ethyl acetate fraction
BHT: Butylated hydroxytoluene
BHA: Butylated hydroxyanisole
TBHQ: Tert-butyl hydroquinone
PG: Propyl gallate
ME: Methanolic extract
PEF: Petroleum ether fractio
CF: Chloroform fraction
SY: Shuangyou
VB: Victoria Blanc
